# YAP‐TEAD inhibition is associated with upregulation of an androgen receptor mediated transcription program providing therapeutic escape

**DOI:** 10.1002/2211-5463.13901

**Published:** 2024-09-19

**Authors:** Roberto Alva‐Ruiz, Ryan D. Watkins, Jennifer L. Tomlinson, Jennifer A. Yonkus, Amro M. Abdelrahman, Caitlin B. Conboy, Erik Jessen, Nathan W. Werneburg, Hendrien Kuipers, Jack W. Sample, Gregory J. Gores, Sumera I. Ilyas, Mark J. Truty, Rory L. Smoot

**Affiliations:** ^1^ Division of Hepatobiliary & Pancreas Surgery, Department of Surgery Mayo Clinic Rochester MN USA; ^2^ Division of Medical Oncology, Department of Oncology Mayo Clinic Rochester MN USA; ^3^ Division of Biomedical Statistics and Informatics, Department of Research Services Mayo Clinic Rochester MN USA; ^4^ Division of Gastroenterology & Hepatology, Department of Medicine Mayo Clinic Rochester MN USA; ^5^ Department of Immunology Mayo Clinic Rochester MN USA; ^6^ Department of Biochemistry and Molecular Biology Mayo Clinic Rochester MN USA

**Keywords:** bile duct tumors, enzalutamide, fibroblast growth factor receptor, Hippo pathway

## Abstract

Cholangiocarcinoma (CCA) is a highly aggressive form of liver cancer and is an increasing cause of cancer‐related death worldwide. Despite its increasing incidence globally and alarming mortality, treatment options for CCA have largely remained unchanged, stressing the importance of developing new effective therapies. YAP activation is common in CCA, and its major transcriptional signaling partners are the TEAD proteins. CA3 is a small‐molecule YAP‐TEAD disrupter discovered utilizing a TEAD reporter assay. Utilizing CCA, gastric cancer cell lines, and patient‐derived xenograft models (PDX), we demonstrate that CA3 is effective in inducing cell death and delaying tumor growth in both *FGFR2* fusion and wild‐type models. CA3 was associated with on‐target decreases in YAP‐TEAD target gene expression, TEAD reporter activity, and overall TEAD levels. Hippo pathway signaling was not altered as there was no change in YAP phosphorylation status in the cells exposed to CA3. RNA sequencing of gastric cancer and CCA models demonstrated upregulation of an androgen receptor–mediated transcriptional program following exposure to CA3 in five unique models tested. Consistent with this upstream regulator analysis, CA3 exposure in CCA cells was associated with increased AR protein levels, and combinatorial therapy with CA3 and androgen receptor blockade was associated with increased cancer cell death. CA3 behaves functionally as a YAP‐TEAD disrupter in the models tested and demonstrated therapeutic efficacy. Exposure to CA3 was associated with compensatory androgen receptor signaling and dual inhibition improved the therapeutic effect.

## Graphical abstract

YAP activation is common in cholangiocarcinoma and its major signaling partners are the TEAD proteins. YAP‐TEAD disruption utilizing CA3 results in cell death and delayed tumor growth *in vitro* and *in vivo*. CA3 exposure was associated with compensatory androgen receptor signaling and combined inhibition improved the therapeutic effects of CA3.

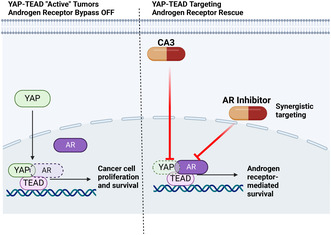

**Abbreviations**
AR: androgen receptor
CCA: cholangiocarcinoma
IF: immunofluorescence
PDX: patient‐derived xenograft
SNV: single nucleotide variants
YAP: yes‐associated protein

Cholangiocarcinoma (CCA) is an aggressive and lethal form of liver cancer with a rising incidence. Despite its increasing incidence and dismal overall survival, treatment for most patients remains largely ineffective, highlighting the need for novel therapeutics and treatment strategies [1, 2, 3, 4]. Targeted therapies, such as FGFR and IDH inhibitors, have shown some promise in a highly selected subset of patients [5, 6]. However, primary and acquired resistance is high even in these selected patients [7, 8]. The addition of immunotherapy to first‐line systemic regimens was recently evaluated in two separate phase III trials [9, 10]. There was modest improvement in median survival to just over 1 year in these studies.

The Hippo‐YAP signaling pathway, among other functions, is important in the regulation of organ growth and cell proliferation and has been implicated in the development of CCA [11, 12]. This pathway is regulated through a series of serine/threonine kinases, which in turn regulate yes‐associated protein (YAP), a coactivator for several transcription factors [13]. When the Hippo pathway is inactive, YAP enters the nucleus where it binds to various transcription factors, including the TEAD family proteins, it then acts as a transcriptional coactivator of genes involved in regulating proliferation, apoptosis, and differentiation [14]. YAP nuclear accumulation with subsequent downstream transcription of target genes has been associated with decreased survival in several cancer types including CCA [15, 16, 17]. YAP as a druggable target is therefore of great interest but has historically been difficult to target given the Hippo pathway's lack of a dedicated cell‐surface receptor and the negative regulatory activity of the pathway itself [15, 16]. Thus, indirect targeting has been evaluated utilizing several approaches [18, 19]. However, more recently YAP‐TEAD disrupters have been described and are starting to undergo preclinical and clinical evaluation [20, 21].

Utilizing a TEAD‐luciferase reporter in 293T cells as a screen for YAP‐TEAD inhibition CA3 was recently validated as a YAP‐TEAD inhibitor [17]. In the initial studies, CA3 demonstrated high levels of inhibitory activity against YAP‐overexpressing cell lines and could synergize with 5FU to limit cancer tumor cell proliferation [17]. The compound has been evaluated in both *in vitro* and *in vivo* models of various malignancies, including esophageal adenocarcinoma, mesothelioma, and epidermal squamous cell carcinoma [17, 22, 23]. Given these promising results, we postulated that CA3 exposure would deliver similar effects in CCA, a cancer with high levels of YAP activation [24]. Utilizing both *in vitro* and *in vivo* models of CCA, herein we demonstrate that CA3, a YAP‐TEAD inhibitor, demonstrates a strong inhibitory effect on YAP‐TEAD transcription, is associated with a decrease in TEAD levels, and can arrest tumor growth in an FGFR2‐fusion patient‐derived xenograft (PDX) model of CCA. We further observed that FGFR2 fusion models, including gastric cancer cell lines, were sensitive to CA3 inhibition, and surprisingly, CA3 exposure was associated with upregulation of an androgen receptor associated transcriptional signature in the tested CCA and gastric cancer models. This upregulation provided therapeutic escape such that simultaneous inhibition of androgen receptor improved therapeutic response to CA3.

## Materials and methods

### Cells and reagents

The human cholangiocarcinoma cell lines HuCCT1 (RRID:CVCL_0324) and RBE (RRID:CVCL_4896) and human gastric cancer cell lines, KATO III (RRID:CVCL_0371) and SNU‐16 (RRID:CVCL_0076), were utilized. All cell lines including HuCCT1, RBE, KATO III, and SNU‐16 were obtained from ATCC (Manassas, VA, USA) and authenticated utilizing short tandem repeat (STR) analysis where multiplex polymerase chain reaction is used to amplify DNA fragments to create a unique genetic cell line profile. These are then compared with ATCC's internal database and Expasy database. Additionally, all cell lines were tested and confirmed to be free of mycoplasma. These processes were completed in the previous 3 years. HuCCT1 and RBE were maintained with Dulbecco's modified Eagle's medium (DMEM) with 10% fetal bovine serum (FBS), 1% penicillin–streptomycin, and 0.2% Primocin™. KATO III was maintained with Iscove's Modified Dulbecco's Medium (IMDM) supplemented with 10% FBS, 1% penicillin–streptomycin, and 0.2% Primocin™ while SNU‐16 was maintained with Roswell Park Memorial Institute Media 1640 (RPMI) supplemented with 10% FPS, 1% penicillin–streptomycin, and 0.2% Primocin™. Cell lines were stored at 37 °C in a 5% CO_2_‐humidified incubator. CA3 (CIL56) and IAG933 were purchased from Selleckchem (Houston, TX, USA). Primary antibodies utilized for immunoblot analysis included: actin (C‐11, polyclonal, sc‐1615; Santa Cruz Biotechnology, Dallas, TX, USA), total YAP (63.7, monoclonal, sc‐271134; Santa Cruz Biotechnology), phospho YAP^s127^ (ab226760, polyclonal; Abcam, Cambridge, UK), anti‐AR (D6F11, monoclonal; Cell Signaling Technology, Danvers, MA, USA), and pan‐TEAD (D3F7L, monoclonal; Cell Signaling Technology). Total YAP (63.7, monoclonal; Santa Cruz Biotechnology) was used for immunofluorescence (IF). For nuclear staining, ProLong Antifade with 4′,6‐diamidino‐2‐phenylindole (DAPI; Life Technologies, Waltham, MA, USA) was used.

### Assessment of cell viability

Cell viability was assessed and IC50 curves generated using CellTiter‐Glo^®^ (CTG) Luminescent Cell Viability Assays (Promega, Madison, WI, USA). HuCCT1, RBE, and KATO III cells were seeded in triplicate in 96‐well plates at a density of 5.0 × 10^3^ cells per well. After 24 h, cells were treated with varying concentrations of CA3 for 72 h. CTG solution was added to each well and luminescence determined using a BioTek Synergy™ H1 (Agilent, Santa Clara, CA, USA) microplate reader. IC50 curves were plotted using graphpad prism version 9.1.0 (San Diego, CA, USA). All assays were repeated at least three times.

### Assessment of cellular death and apoptosis

Cell lines were seeded in 96‐well plates at 5.0 × 10^3^ cells/well for 24 h. Cells were seeded in triplicate. Each cell line was then exposed to 1 μm of CA3 for 24 h. Cells were then incubated with propidium iodide (PI) 2 μg·mL^−1^ and Hoechst 5 μg·mL^−1^ for 30 min at 37 °C, washed and then imaged using a Celigo Image Cytometer (Nexcelom Bioscience, Lawrence, MA, USA). Cell death was plotted using graphpad prism version 9.1.0. The apoptotic effects of CA3 were measured using Caspase‐Glo 3/7 reagent from Promega. Cells from each cell line were counted and plated in triplicate in 96 well plates at a concentration of 5 × 10^3^ cells per well. Each cell line was treated with 1 μm of CA3 for 24 h. Cell suspensions were gently mixed in a 1 : 1 ratio with Caspase‐Glo 3/7 assay reagent. Plates were covered and placed on a shaker for 45 min to ensure mixture of cells with reagent. Fluorescence was measured using BioTek Synergy™ H1 microplate reader.

### Quantitative reverse transcription PCR (RT‐qPCR) and immunoblot analysis

Total RNA was isolated from cells and tumor tissue using Trizol Reagent (Ambion, Waltham, MA, USA). Two micrograms of RNA was converted to cDNA using M‐MLV Reverse Transcriptase (Invitrogen, Waltham, MA, USA) and random primers. cDNA was then analyzed using real‐time PCR (Light Cycler 480 II; Roche Diagnostics, Indianapolis, IN, USA) to quantify YAP target genes. SYBR® Green (Roche Diagnostics) was used as the fluorescent reporter molecule. Target gene expression was normalized to 18 S and quantification performed per previously described methods [16]. Three technical replicates were performed for each run, and a minimum of three biologic replicates were completed for each cell line and/or tumor tissue. The primers used can be seen in Table S1. Whole‐cell lysates were collected using lysis buffer containing protease inhibitors (Roche Diagnostics), phosphatase inhibitors (Roche Diagnostics), and 1 mmol·L^−1^ of PMSF. Protein concentration was measured using Bradford reagent (Sigma‐Aldrich). Proteins were resolved with SDS/PAGE, transferred to a nitrocellulose membrane, and subsequently incubated with primary antibodies overnight at 4 °C in 5% BSA‐TBS Tween. Membranes were then washed for 1 h in TBS Tween and then secondary antibodies were added at a concentration of 1 : 5000 and incubated for 1 h at room temperature. Blots were visualized with enhanced chemiluminescence (ECL).

### TEAD reporter assay

HuCCT1 cells were transduced with a lentiviral TEAD‐dependent firefly luciferase reporter (#79833; BPS Bioscience, San Diego, CA, USA) and constitutive Renilla luciferase reporter (#79565; BPS Bioscience) and selected with puromycin. Firefly and Renilla luciferase luminescence were measured using the Dual Luciferase Assay system (BPS Bioscience) according to manufacturer protocol. Firefly luminescence was first normalized to Renilla luminescence as an internal control. Cells were treated with CA3 at 0.500 μm for 24 h prior to measurement of firefly luciferase activity.

### Immunohistochemistry and YAP immunofluorescence staining

Characterization of each xenograft model was done using standard immunohistochemistry (IHC) and performed by the Pathology Research Core (Mayo Clinic, Rochester, MN, USA) using the Leica Bond RX stainer (Leica, Deer Park, IL, USA). The following antibodies were used: HepPar1 (1 : 100, clone OCH1ES; Abcam) and CK7 (1 : 400, clone OV‐TL 12/30; Dako, Santa Clara, CA, USA).

### *In vivo* xenograft mouse model

All *in vivo* studies were approved by the Institutional Animal Care and Use Committee at Mayo Clinic (A00003954‐18‐21). One histologically validated patient derived xenograft (PDX) was developed as previously described by our group (Fig. S1B) [25]. At the time of surgical resection, patient tumor tissue is examined for viability. Once viable cancerous tissue is confirmed, tissue is placed into transport tubes containing cooled tissue culture media (Roswell Park Memorial Institute, RPMI; Invivogen, Carlsbad, CA, USA) and subsequently implanted into the flanks of NOD‐SCID mice. Tumor volume and body weight were measured at treatment study start and subsequently recorded every 7 days. CA3 was injected intraperitoneally in the treatment group at 1.5 mg·kg^−1^ per mouse three times per week for 3 weeks. Vehicle mice were given 100 μL intraperitoneal injections of PBS three times per week throughout the duration of study. The formula for tumor volume was calculated as (*L* × *W* × *W*)/2. Change in tumor volume was determined as change from baseline and subsequently represented as a fold change from baseline measurement at treatment start.

### Bioinformatics

#### RNA‐sequencing analysis

The raw RNA sequencing paired‐end reads for the samples were processed through the Mayo RNA‐Seq bioinformatics pipeline, map‐rseq version 3.1.4 [26]. Briefly, map‐rseq employs the very fast, accurate and splice‐aware aligner, star, to align reads to the reference human genome build hg38 [27]. The aligned reads are then processed through a variety of modules in a parallel fashion. Gene and exon expression quantification are performed using the Subread package to obtain both raw and normalized (FPKM—Fragments Per Kilobase per Million mapped reads) reads [28]. star fusion algorithm is used to identify and report any expressed gene fusions in the samples [27]. Likewise, expressed single nucleotide variants (SNVs) and small insertions‐deletions (indels) are detected using a combination of bioinformatics tools such as GATK, Haplotype caller and RVBoost [29, 30]. Known and novel gene isoforms are assembled and quantified using stringtie to enable detection of alternative spliced isoforms. In addition, differential exon usage is evaluated using DEXSeq to enable comparison across conditions for alternative splicing at the exon level [31, 32]. Finally, comprehensive analyses are run on the aligned reads to assess quality of the sequenced libraries. Results from all modules described above are linked through a single html document and reported by map‐rseq.

#### Differential expression

Using the raw gene counts report from map‐rseq, genes that are differentially expressed between the groups were assessed using the bioinformatics package edger 2.6.2 [33]. Genes found different between the groups were reported along with their magnitude of change (log_2_ scale) and their level of significance (False Discovery Rate, FDR < 5%).

#### Pathway analysis

Using differentially expressed genes, canonical pathway analysis was performed using the Ingenuity pathway analysis software ipa (Ingenuity® Systems, www.ingenuity.com). Biological functions and diseases information within the ipa software will be used to investigate canonical pathways of interest.

#### Upstream transcription factor and kinase prediction

CA3 treated and vehicle samples were directly compared for all five cell lines individually to obtain a list of genes with either positively or negatively changing expression. Each gene list was submitted for transcription factor and subsequent kinase prediction using the eXpression2Kinases (x2k) tool. The top significantly enriched transcription factors and kinases were compared to identify commonalities between cell lines. Each cell line was analyzed separately, and significance reported as the −log*P*value from the x2k tool. The significance data plotted in Fig. 5B and Fig. S3A represent the average of these significant values.

### Statistical analysis

Statistical tests, including Student's *t*‐tests, were performed using graphpad prism version 9.1.0. A *P*‐value of < 0.05 was considered significant.

## Results

### CA3 exposure decreases cell viability and sensitizes CCA cell lines to standard of care first‐line chemotherapy

**Fig. 1:**
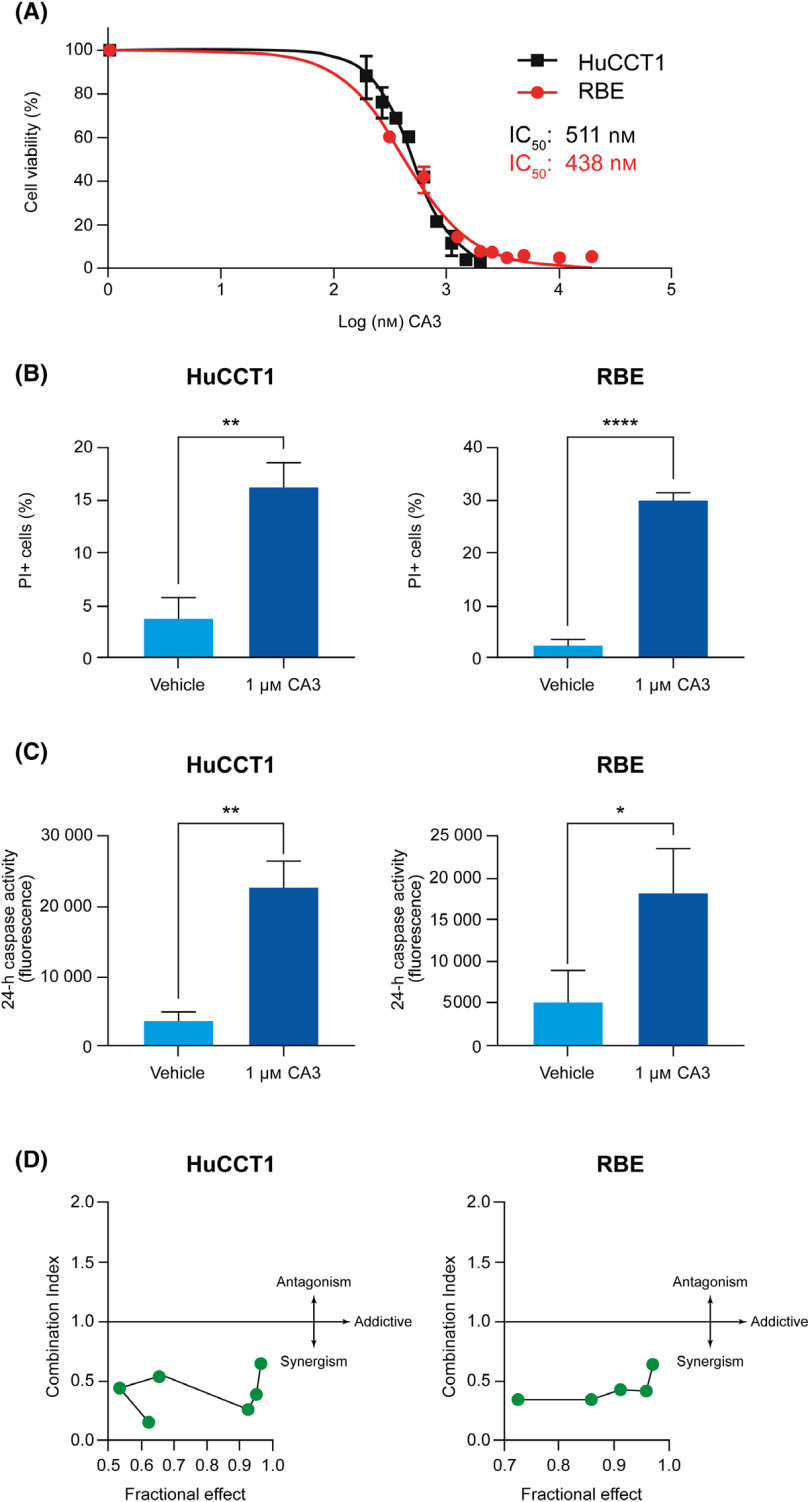
CA3 exposure decreases cell viability via apoptosis in CCA cells lines. (A) HuCCT1 and RBE cells were treated with CA3 at doses ranging from 300 to 20 000 nm. Half maximal inhibitory concentrations were determined with a short‐term viability assay, Cell Titer Glo. (B) Cell death was evaluated after 24 h of CA3 treatment by staining with propidium iodide in HuCCT1 and RBE cells. (C) HuCCT1 and RBE cells show increased apoptosis as measured by Caspase 3/7‐Glo assays after 24‐h treatment with CA3. (D) Combinatorial indices of HuCCT1 and RBE cells treated with increasing concentrations of CA3 and gemcitabine/cisplatin. Combination treatment demonstrated synergy in both cell lines. Data are shown as mean ± SEM (*P < 0.05, **P < 0.005, ****P < 0.0001). Statistical analysis was performed with two‐tailed Student's *t‐*test.

Two cholangiocarcinoma cell lines, HuCCT1 and RBE, were treated with CA3 at varying concentrations to determine the effects on cell viability. As shown in Fig. 1A, CA3 inhibited the growth of both cell lines in a concentration‐dependent manner. Inhibition was accomplished at low concentrations of CA3, with half maximal inhibitory concentration (IC_50_) doses of 511 nm (438.1–540.4 nm) and 438 nm (402.4–473.3 nm) identified for HuCCT1 and RBE cell lines respectively at 72 h. Cell death was confirmed with propidium iodide staining (Fig. 1B) and was due to apoptosis as shown through increased capsase‐3 and ‐7 activity (Fig. 1C). Additionally, we exposed each cell line to increasing concentrations of CA3 (0.125–2 μm) and gemcitabine/cisplatin (0.00125–0.005 μm) and assessed the combinatorial effects with the calcusyn software (Biosoft, Cambridge, UK). All combination indices were < 1 suggesting synergy. Combination indices of HuCCT1 cells were 0.155, 0.446, 0.550, 0.265, 0.393, and 0.641. Combination indices of RBE cells were 0.348, 0.339, 0.427, 0.417, and 0.639 (Fig. 1D).

### CA3 decreases YAP‐TEAD cotranscriptional activity *in vitro*

**Fig. 2:**
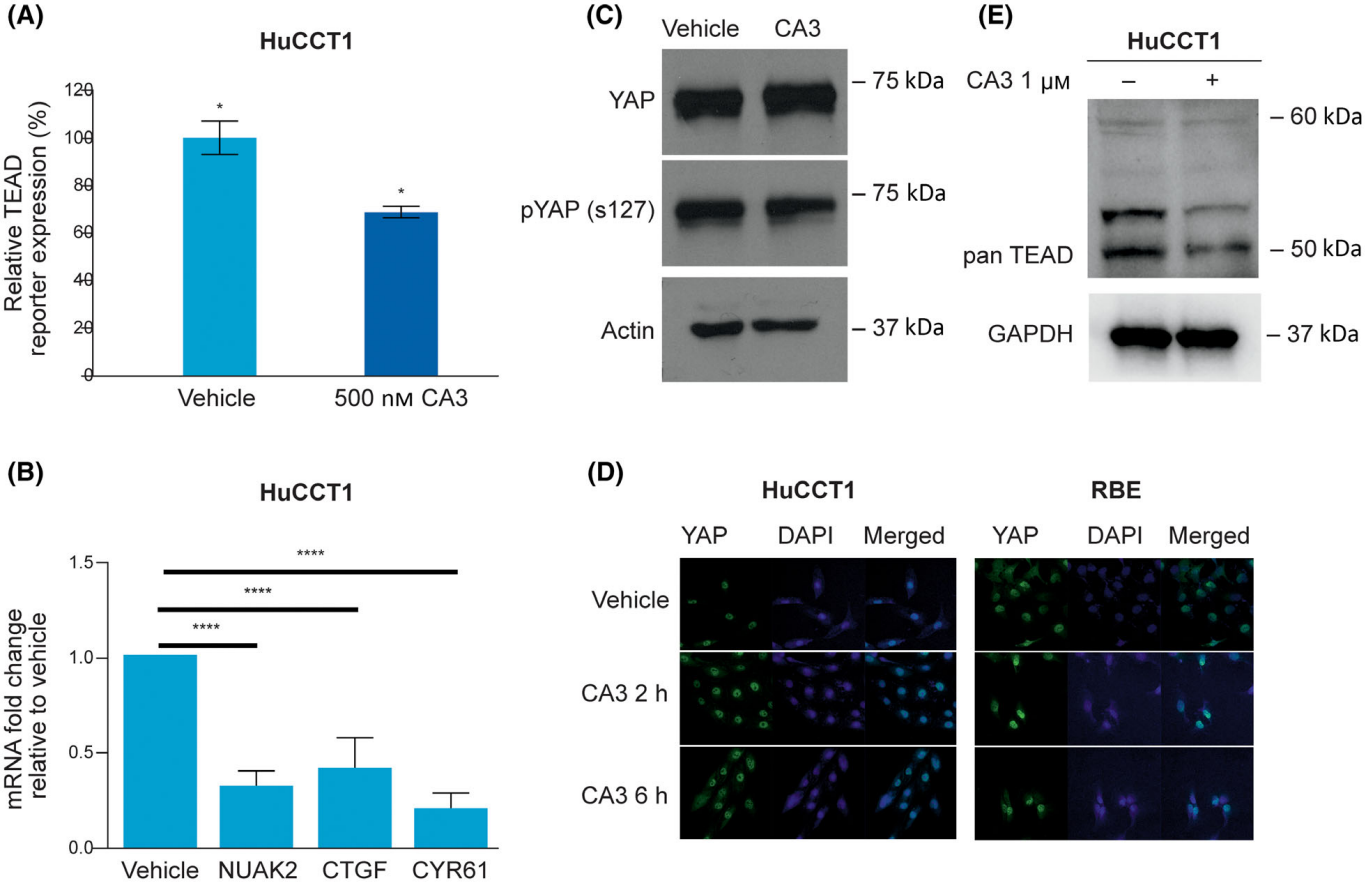
CA3 decreases YAP‐TEAD co‐transcriptional activity *in vitro*. (A) Luciferase reporter assay for TEAD is decreased after treatment with CA3 (0.500 μm, 24 h). Relative TEAD reporter expression is depicted as mean ± standard deviation. (B) mRNA expression in HuCCT1 cells treated with CA3 (5 μm, 24 h). Fold expression change to vehicle is depicted as mean ± standard deviation. (C) HuCCT1 cell lysates were treated with 1 μm of CA3 and subjected to immunoblot for total YAP and phospho‐YAP^S127^. Levels of both total YAP and serine 127 phosphorylation of YAP were unchanged. (D) HuCCT1 and RBE cells were subjected to CA3 (1 μm, 6 h) and immunofluorescence for YAP was undertaken, representative images are displayed for vehicle (top) versus treated cells (bottom). YAP was predominantly nuclear regardless of CA3 exposure or dose. (E) HuCCT1 cell lysates were treated with 1 μm CA3 and subjected to immunoblot for TEAD levels. CA3 exposure was associated with a decrease in TEAD levels. Data are shown as mean ± SEM (*P < 0.05; ****P < 0.0001). Statistical analysis was performed with two‐tailed Student's *t‐*test.

The YAP‐TEAD transcriptional activity and effects of CA3 were then evaluated by TEAD reporter assay as well as RT‐PCR for canonical YAP‐TEAD target genes. HuCCT1 cells transduced with a lentiviral TEAD‐dependent firefly luciferase reporter and constitutive Renilla luciferase reporter demonstrated a significant decrease in YAP‐TEAD transcription after exposure to CA3 (Fig. 2A). Additionally, mRNA expression of canonical YAP target genes including NUAK2, CTGF, and CYR61 were significantly decreased in CA3‐treated cells (Fig. 2B). The mechanisms of YAP‐TEAD inhibition by CA3 are not fully defined as it was validated as a YAP‐TEAD inhibitor in a TEAD‐reporter screen [22]. It has been suggested previously that CA3 can directly decrease YAP levels [17, 22]. We evaluated the mechanism of decreased YAP‐TEAD transcriptional activity in the CCA cells by first evaluating the total YAP levels and the serine 127 phosphorylation of YAP (a marker of Hippo pathway activity) by immunoblot. In the HuCCT1 CCA cell line, total YAP did not change and S127‐YAP levels did not increase following exposure to CA3, suggesting no direct effects on Hippo signaling or YAP itself (Fig. 2C). Furthermore, we assessed YAP subcellular localization before and after exposure to CA3. Consistent with a model by which YAP itself is not altered in level or regulation by CA3, we noted that at baseline CCA cell lines had predominately nuclear YAP localization, which was not altered by CA3 administration, even at high concentrations (Fig. 2D). We then evaluated TEAD levels utilizing a pan‐TEAD antibody by immunoblot. We noted that CA3 exposure was associated with a decrease in TEAD levels. These findings suggest that CA3 can decrease YAP‐TEAD transcriptional activity in CCA cells, at least in part by reducing TEAD levels (Fig. 2E).

### FGFR2 altered models are sensitive to CA3

**Fig. 3:**
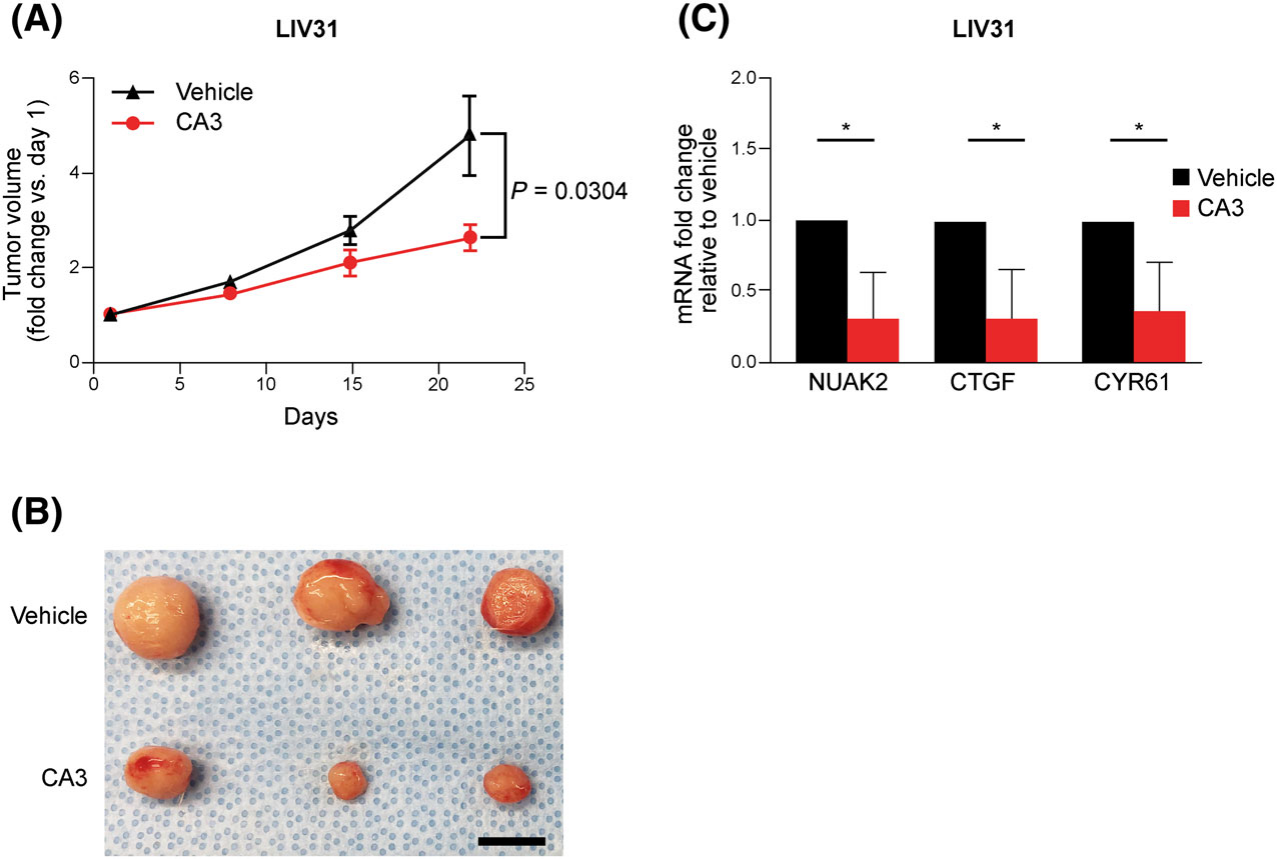
CA3 arrests tumor growth in an *in vivo* model of CCA. (A) Change in tumor volume for vehicle‐treated vs. CA3‐treated mice in LIV31 PDX tumors (1.5 mg·kg^−1^, 3 weeks). Treated mice demonstrated significantly decreased tumor growth. Total tumor weight was considered and not normalized to mouse body weight. (B) Representative images of LIV31 vehicle‐treated (top row) and CA3‐treated tumors at study end. Seven vehicle mice and seven CA3 treated mice were utilized in the study. Scale bar: 1 cm. (C) NUAK2, CTGF, and CYR61 mRNA expression levels were decreased in CA3 treated LIV31 tumor tissue. mRNA levels are expressed as fold change relative to vehicle. Seven tumors, each from vehicle and CA3 treated were utilized to perform analysis. Data are shown as mean ± SEM (*P < 0.05). Statistical analysis was performed with two‐tailed Student's *t‐*test.

**Fig. 4:**
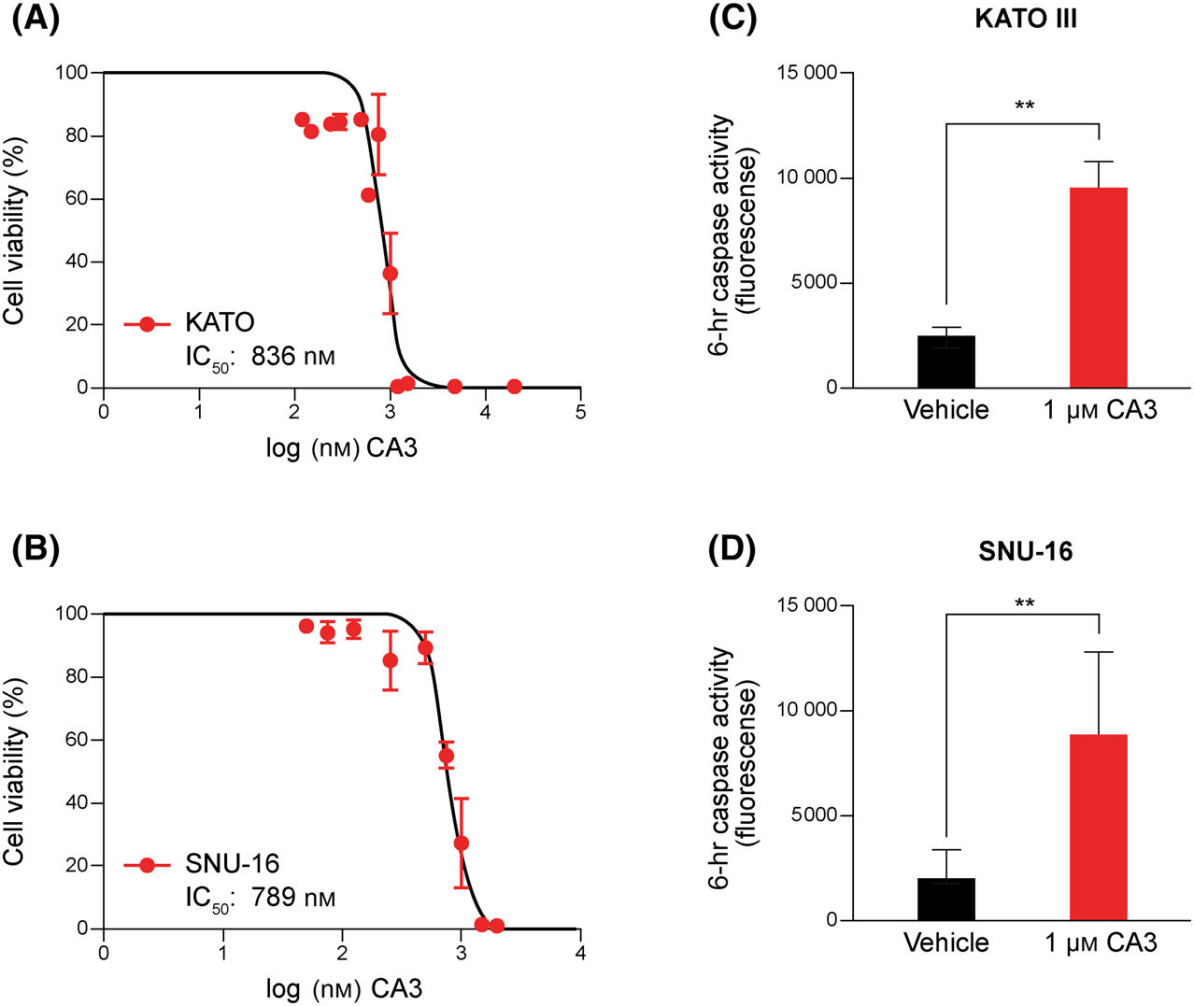
FGFR2 gastric cancer cell lines are sensitive to CA3 exposure. (A, B) KATO III and SNU‐16 cells were exposed to increasing concentrations of CA3. Half maximal inhibitory concentrations were determined with Cell Titer Glo. (C, D) KATO III and SNU‐16 cells showed increased apoptosis as measured by Caspase 3/7‐Glo assays after 6 h of treatment with CA3. Data are shown as mean ± SEM (**P < 0.005). Statistical analysis was performed with two‐tailed Student's *t‐*test.

We previously demonstrated that CA3 was effective *in vivo* in an FGFR2 fusion‐positive patient‐derived xenograft model of CCA [19]. This previous work suggested that FGFR2 fusion models were sensitive to YAP‐targeted therapies as single agents. We expanded this previous work by evaluating CA3 in an additional FGFR2‐fusion PDX model. LIV31 a histologically validated patient‐derived xenograft model was utilized as a CCA model. Clinical details including FGFR2 fusion status, anatomic subtype, age at resection, and sex can be seen in Fig. S1A. NOD/SCID mice bearing LIV31 flank tumors were given CA3 at a dose of 1.5 mg·kg^−1^ by intraperitoneal injection three times per week for 3 weeks. CA3‐treated models demonstrated significantly decreased tumor growth as compared to tumors in the vehicle‐treated control mice (Fig. 3A). Overall, CA3 treatment was well‐tolerated, with minor weight loss observed in vehicle‐treated and CA3‐treated mice (Fig. S2). Representative images of LIV31 vehicle‐treated and CA3‐treated tumors at study end can be seen in Fig. 3B. Next, we examined the mRNA expression levels of YAP target genes NUAK2, CTGF, and CYR61 and compared them with vehicle‐treated mice. mRNA expression levels of NUAK2, CTGF, and CYR61 were significantly decreased in CA3‐treated tumor tissue vs vehicle‐treated mice in LIV31 (Fig. 3C) [34]. Given these findings, we questioned whether other FGFR2 fusion tumor models might be sensitive to CA3. While the HuCCT1 cell line carries a *KRAS* (G12D) mutation and the RBE line carries an *IDH1* (R132S) mutation, there are no commercially available FGFR2‐altered CCA cell lines. Alternatively, we obtained the FGFR‐altered gastric cancer cell lines KATOIII (*FGFR2‐ULK4* fusion) and SNU‐16 (*FGFR2‐PLPP4* fusion). These cell lines were treated with increasing concentrations of CA3 and demonstrated sensitivity with IC50 of 836 and 789 nm (Fig. 4A,B). The effect was due to apoptosis activation as evidenced by increased caspase 3/7 activity (Fig. 4C,D).

### CA3 exposure is associated with upregulation of an androgen receptor associated transcriptomic program

**Fig. 5:**
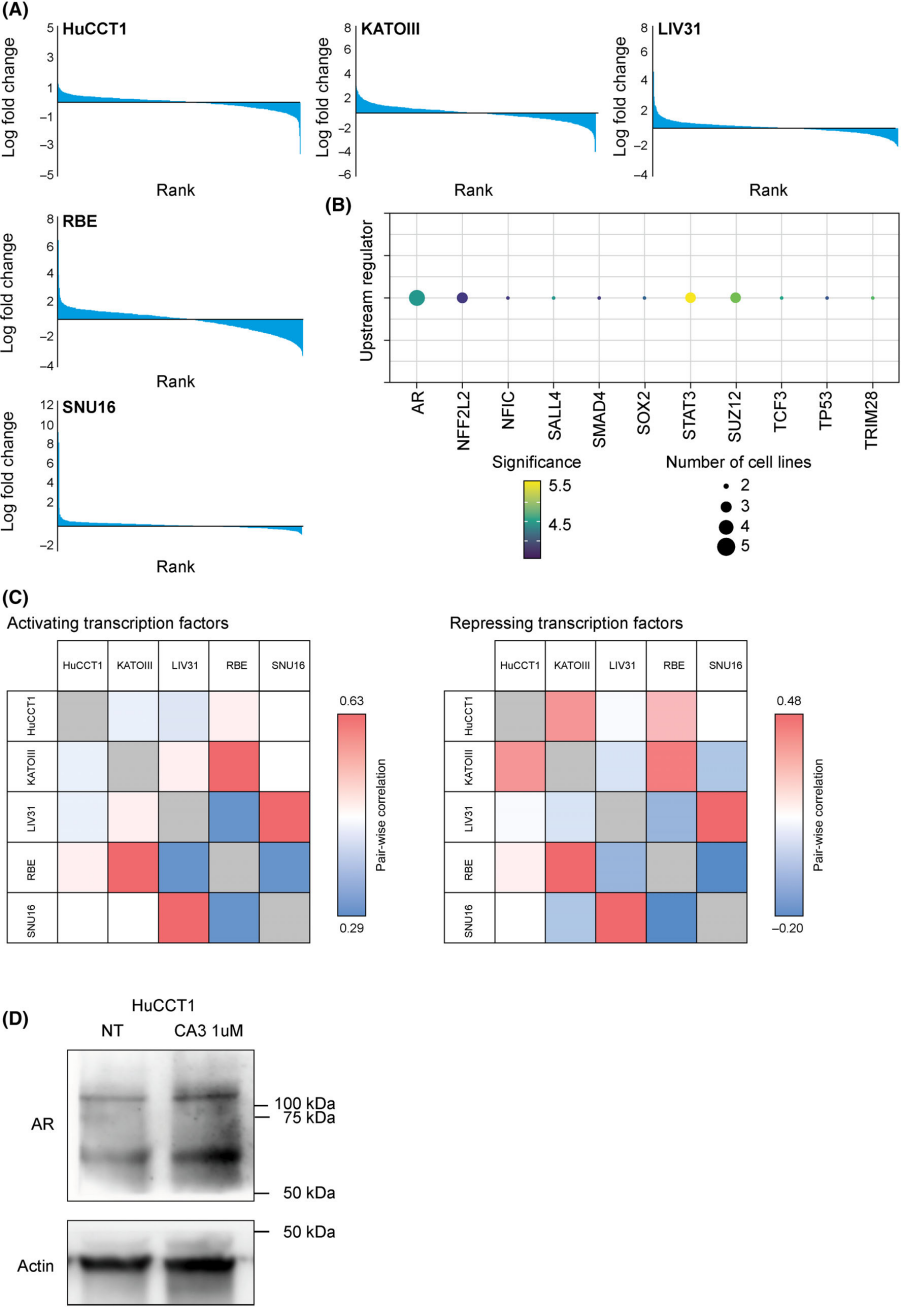
Transcriptional upstream regulators of CA3 treatment across cell lines. (A) Distribution of log fold changes of genes for each cell type ordered by rank. (B) Recurrence (size of dot, number of cell lines) and significance (color of dot, −log*P*value) of upstream regulators predicted for differential genes between cell lines. (C) Pair‐wise correlation between cell lines for significance of activating or repressing transcription factors. (D) HuCCT1 cells were subjected to CA3 (1 μm, 24 h) and immunoblot for androgen receptor performed. Treated cells demonstrated increased levels AR.

In order to try to understand the effects of CA3 in the tested cholangiocarcinoma and gastric cancer models broadly, we then sought to determine the global transcriptomic changes via RNA sequencing. We compared CA3 treatment to vehicle in the four cell lines (HuCCT1, RBE, KATOIII, and SNU16) and in the LIV31 CCA PDX tumor tissue. In these models, we noted alteration of gene expression of CA3‐treated cells compared with empty vehicle resulting in 99 (HuCCT1), 328 (KATOIII), 213 (LIV31), 663 (RBE), and 75 (SNU16) genes with an absolute log fold change > 2. Based on the proposed mechanism of CA3, we then asked what upstream and downstream regulators were associated with the altered genes. Notably, transcription factors predicted to regulate either the positive or negative log fold differences in gene expression (Fig. 5A) between CA3‐treated and empty vehicle cells clustered independent of cholangiocarcinoma or gastric origin (Fig. 5C). Instead, KATOIII correlated best with RBE, and LIV31 correlated best with SNU16. Androgen receptor (AR) was the common transcription factor noted as an upstream regulator for positively regulated genes between all five models treated with CA3 (adjusted *P*‐value < 0.05), such that exposure to CA3 was associated with an increased AR transcriptome in all five models. Additionally, SUZ12, NFE2L2, and STAT3 were each identified as upstream regulators in three of the cell lines, and TCF3, TP53, and SOX2 were identified in two of the cell lines (Fig. 5B). Less consensus was observed in transcriptional regulators for the down regulated genes; with SMC3 in three models, and ZEB1 and BCL3 in two (Fig. S3A). In the RNA sequencing dataset, YAP mRNA levels appeared stable across all models while TEAD 1–4 were highly variable across models. Comparisons between each sample were performed. Biologic replicates were not performed (Fig. S3B). Given the upregulation in AR‐regulated genes we evaluated AR levels by immunoblot in HuCCT1 cells exposed to CA3. Consistent with the signature noted in the RNA sequencing analysis, we observed an increase in AR levels at a protein level (Fig. 5D). We exposed HuCCT1 cells to a second, bonafide pan‐TEAD inhibitor IAG933 [35]. Similar to the CA3 response, after 24 h we noted an increase in AR protein levels by immunoblot (Fig. S3C).

### Androgen receptor blockade improves response to CA3

**Fig. 6:**
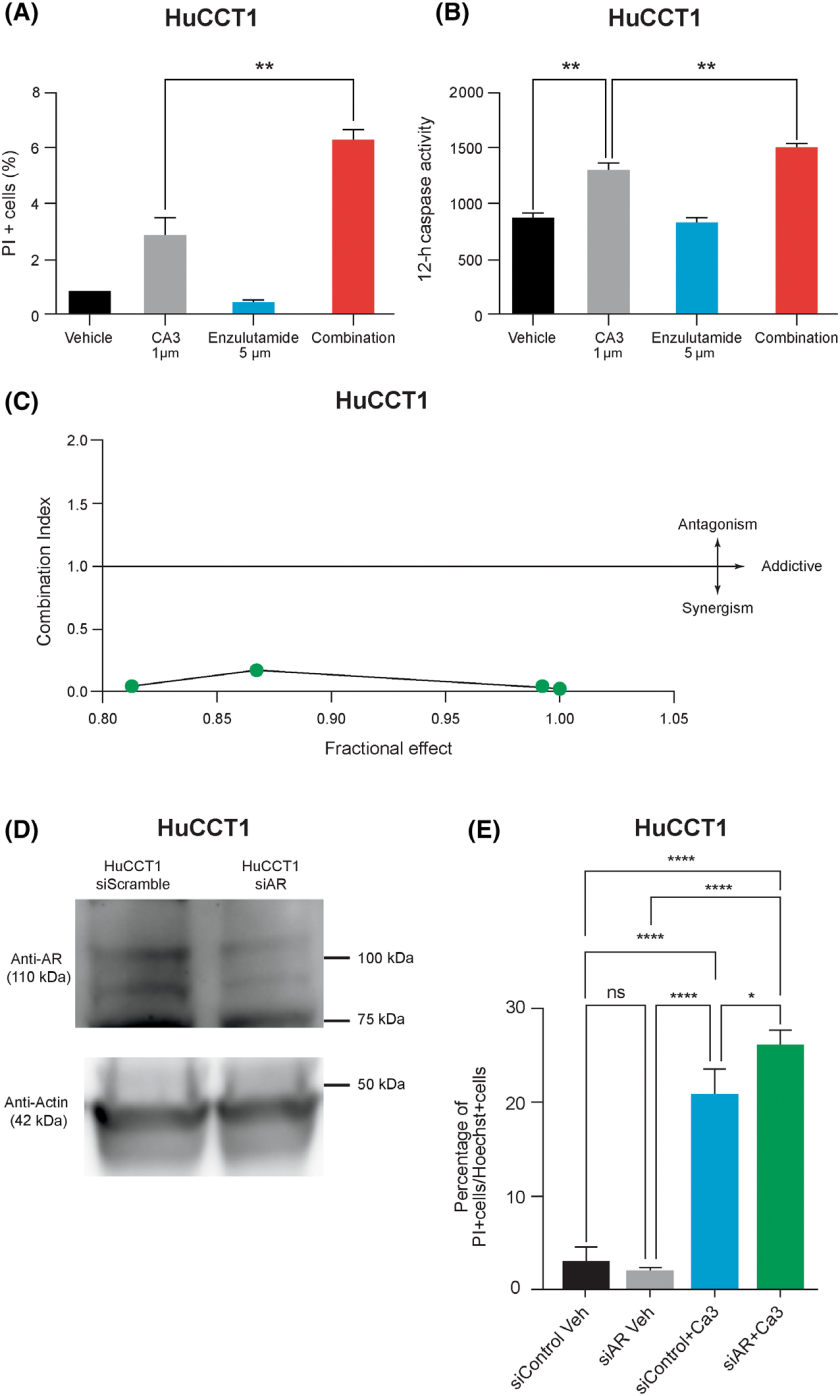
Androgen receptor inhibition improves response to CA3. (A, B) HuCCT1 cells were exposed to CA3, enzalutamide, and combination therapy. Cell death was significantly higher with combination therapy versus either drug alone as demonstrated with propidium iodide staining. Caspase‐3/7 activation was significantly higher with combination therapy. **P < 0.005. (C) HuCCT1 cells exposed to combination treatment with CA3 and enzalutamide demonstrated synergy as demonstrated with combination indices < 1. (D) Immunoblot demonstrating functional knockdown of androgen receptor in HuCCT1 cells. (E) Cell death was significantly higher in siAR knockdown cells treated with CA3 compared to control. Data are shown as mean ± SEM (****P < 0.0001; *P < 0.05; ns, not significant). Statistical analysis was performed with one‐way ANOVA.

Recent data suggest that AR can compete with YAP for TEAD occupancy and signaling [36]. Given that we saw upregulation of androgen receptor‐associated transcriptional programs following exposure to CA3, we next sought to evaluate whether in this context AR signaling may provide therapeutic escape for the CCA cells. We exposed the CCA cell line HuCCT1 to 1 μm CA3 and 5 μm enzalutamide, a clinically available AR inhibitor. We noted an increase in cell death and caspase 3/7 activation in cells exposed to the combination as compared to either drug alone (Fig. 6A,B). We next exposed the cells to a range of concentrations of both inhibitors and assessed the combinatorial effects utilizing calcusyn software. At increasing 1 : 1 doses (1–1000 μm) of CA3 and enzalutamide the combination indices were all < 1 at 0.031, 0.165, 0.014, and 0.013, respectively, indicating a synergistic effect (Fig. 6C). To ensure the effects were specific to AR inhibition we also performed siRNA mediated knockdown of androgen receptor (Fig. 6D) and observed sensitization to CA3 treatment in those cells (Fig. 6E).

### Androgen receptor is expressed in multiple human tumor types

**Fig. 7:**
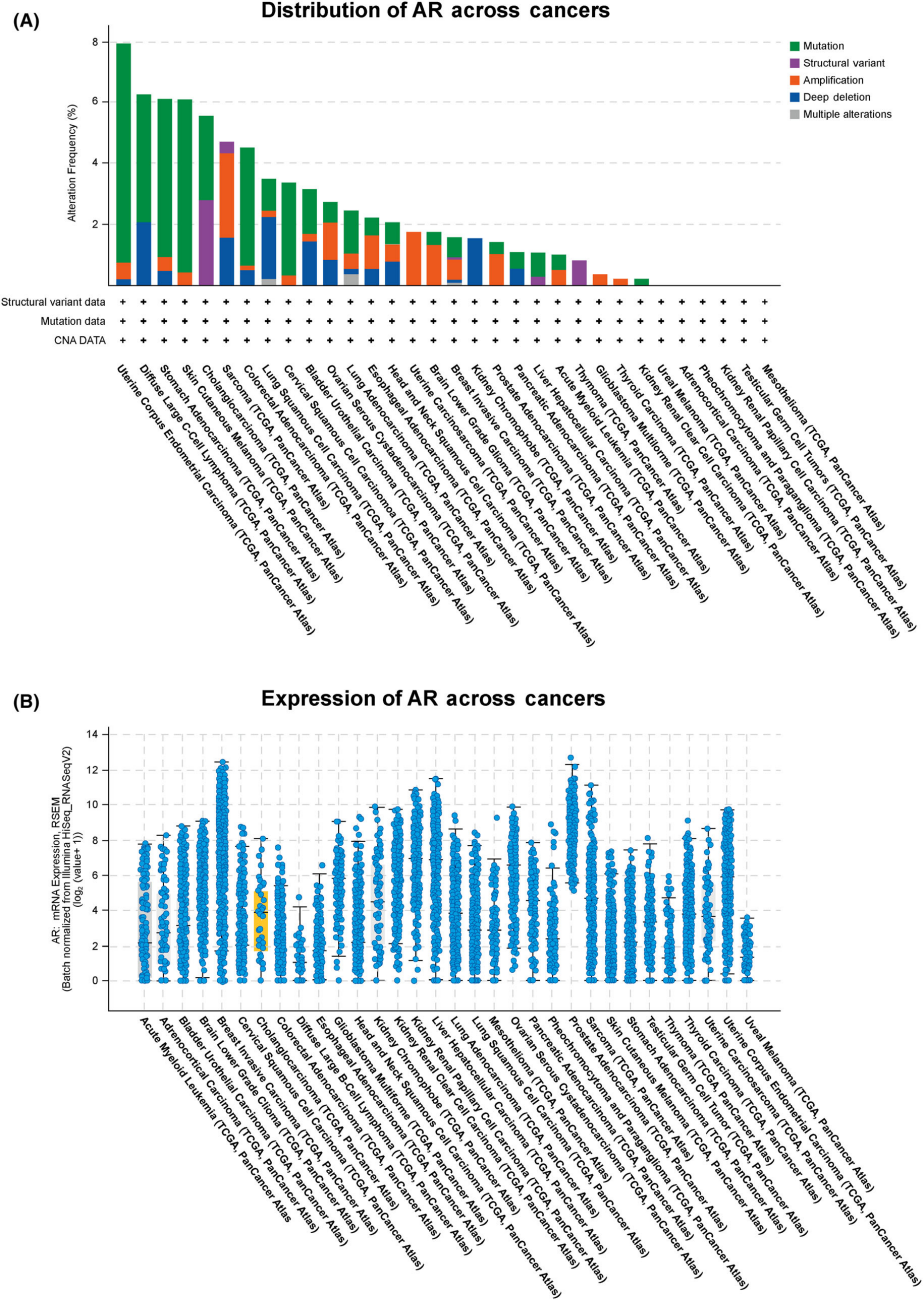
Distribution and expression of androgen receptor (AR) across various cancer types utilizing TCGA Pan‐Cancer Atlas. (A) Cholangiocarcinoma demonstrated the highest number of structural variants out of the 32 cancer types evaluated. (B) mRNA expression of AR across tumor types. AR was expressed in cholangiocarcinoma.

Given the findings from our experimental models, we next evaluated publicly available sequencing datasets to understand the overall expression and alterations in AR. We noted several genetic alterations in AR across multiple tumor types with cholangiocarcinoma demonstrating the highest frequency of structural variants out of the 32 tumor types evaluated (Fig. 7A). Finally, we assessed the abundance of AR at an RNA level. While within individual tumor types there was a broad range of expression, cholangiocarcinoma demonstrated AR expression (Fig. 7B).

## Discussion

This study delineates the efficacy and mechanisms of action of the novel YAP‐TEAD inhibitor CA3 in multiple cancer models. Specifically, the major findings include (a) CCA cell lines are sensitive to CA3 demonstrating a decrease in TEAD levels, (b) FGFR2 fusion CCA and gastric cancer models are sensitive to CA3, (c) exposure to CA3 is associated with upregulation of an AR regulated transcriptional program, and (d) CA3 can synergize with AR inhibition to improve cancer cell killing. These findings suggest that YAP‐TEAD inhibition may have clinical efficacy in selected cancer models and that AR represents a therapeutic escape pathway for YAP‐TEAD inhibition.

YAP‐TEAD inhibition remains of interest in many cancer types and preclinical testing of CA3 activity has been observed in epidermal squamous cell carcinoma, esophageal adenocarcinoma, mesothelioma, and neuroblastoma [17, 22, 23, 37]. YAP‐TEAD‐specific targeting has also been pursued with other compounds. For example, in a recent phase 1 trial that enrolled patients with refractory solid tumors, VT3989, a highly potent and selective inhibitor of TEAD palmitoylation which blocks YAP function, was well‐tolerated and achieved durable RECIST antitumor responses [21]. YAP‐TEAD targeting may also have benefit when used in combinatorial therapy due to the role of YAP‐TEAD signaling in therapeutic resistance mechanisms [38, 39, 40]. For example, recent work has demonstrated that YAP‐TEAD signaling could push cells to treatment‐related dormancy, providing escape from apoptotic signaling. The authors utilized EGFR‐mutant non‐small‐cell lung cancer cells to elucidate the mechanistic underpinnings behind this dormant state. Utilizing next‐generation sequencing, they found that the treatment‐related (EGFR/MEK inhibition) dormant cell state is associated with increased TEAD transcription factor binding, as measured by increased *CTGF* and *ANKRD1* expressions. When these dormant cells were then treated with concomitant EGFR/MEK inhibition and an indirect inhibitor of YAP, the number of dormant cells was dramatically reduced suggesting that the EGFR/MEK inhibition resistance is dependent upon YAP/TEAD signaling [41].

In addition, resistance to a novel KRAS inhibitor was discovered to involve YAP/TAZ signaling which could be overcome utilizing a novel pan‐TEAD inhibitor. The FDA recently approved the drug sotorasib for treatment of KRASG12C‐mutant non‐small‐cell lung cancers. Even in responders, progression‐free survival was limited in the clinical trial, and in patients who later developed treatment resistance no detectable genetic alterations were discovered to be playing a role in treatment resistance [42, 43]. Using cell lines resistant to sotorasib, Hagenbeek et al demonstrated that YAP translocation to the nucleus was increased in sotorasib‐resistant cells compared with control cells and furthermore demonstrated that the open areas of chromatin in sotarasib‐resistant cells were enriched in YAP/TAZ target genes as well as upregulated as compared to control cells treated with sotorasib. Through the discovery of a novel TEAD inhibitor, GNE‐7883, the authors were able to overcome resistance to KRAS12C treatment by inhibiting the downstream transcription program of YAP, TAZ, and TEAD [20]. In our studies, we have noted that *FGFR2* fusion models have been sensitive to YAP‐TEAD directed therapy. Acquired resistance to FGFR2 inhibition is common, and although some mechanisms have been delineated, there remains subsets of patients for whom genetic causes of resistance are not readily evident [44, 45]. The role of YAP‐TEAD signaling in therapeutic escape from FGFR2 inhibition is an important area of future research.

One of the most striking findings from our current study was the recognition that CA3 exposure upregulated an AR‐regulated transcriptional program in all the models tested. Notably, YAP‐TEAD were not identified in the upstream regulator analysis. This analysis involves transcription factor enrichment but then also protein–protein interaction prediction upstream of transcription factors. It is possible that while we noted downregulation of specific YAP‐TEAD target genes following exposure of cell lines to CA3, that the overall signature did not support identification of YAP‐TEAD as primary upstream regulators of the response either due to overall abundance of the transcripts or due to compensatory signaling mechanisms such as AR upregulation. In terms of AR specifically in biliary tract cancer, structural variants have been associated with a risk of gallbladder cancer, and AR signaling has been implicated in hepatocellular cancer development although there has not been a direct link with cholangiocarcinoma [46, 47]. Recently, AR was found to compete with YAP for TEAD occupancy in prostate cancer models [36]. Based on these findings, we hypothesized that AR could be providing therapeutic escape for cells exposed to CA3 and we found that subsequent combinatorial therapy with an AR inhibitor was beneficial. We further showed that AR protein levels are increased *in vitro* cell line of CCA treated with CA3 suggesting that indeed AR upregulation occurs in response to the stress of YAP‐TEAD inhibition. The implications of this competitive signaling for YAP‐TEAD directed therapies will need to be further explored including to understand whether this is a context specific finding or a more globally occurring phenomenon that will require co‐inhibition. Furthermore, the cell survival signaling downstream of AR activation in these cancer models will need to be further elucidated.

Overall, these studies support CA3 as a therapeutic approach in selected CCA models and suggest that combinatorial approaches with AR‐directed inhibitors should be considered in the setting of YAP‐TEAD targeting.

## Conflict of interest

The authors declare no conflict of interest.

### Peer review

The peer review history for this article is available at https://www.webofscience.com/api/gateway/wos/peer‐review/10.1002/2211‐5463.13901.

## Author contributions

RLS conceived the study and designed experiments in conjunction with RA‐R. RA‐R, RDW, JAY, CBC, AMA, JTL, EJ, NWW, JWS, and HK performed experiments. RA‐R, JTL, RDW, EJ, and AMA performed computational analyses and analyzed data. RA‐R and RLS wrote the manuscript. MJT provided expertise with PDX. GJG provided expert review. All the authors approved the manuscript.

## Supporting information


**Fig. S1.** Clinical and histological details of PDX model LIV31.
**Fig. S2.** Weight change between vehicle and CA3 treated LIV31 mice.
**Fig. S3.** Transcription regulators for downregulated genes, TEAD & YAP mRNA levels across cell lines, and immunoblot of AR in HuCCT1 after treatment with IAG933.

**Table S1.** RT‐PCR primers.

## Data Availability

The data that support the findings of this study are available from the corresponding author (smoot.rory@mayo.edu) upon reasonable request.
