# Supplementary material for: YAP‐TEAD inhibition is associated with upregulation of an androgen receptor mediated transcription program providing therapeutic escape
Source: FEBS Open Bio. 2024 Sep 19;14(11):1873–87. doi: 10.1002/2211-5463.13901 (PMC11532981; doi:10.1002/2211-5463.13901)
Supplement: Supplementary file 1 — Fig. S1. Clinical and histological details of PDX model LIV31. Fig. S2. Weight change between vehicle and CA3 treated LIV31 mice. Fig. S3. Transcription regulators for downregulated genes, TEAD & YAP mRNA levels across cell lines, and immunoblot of AR in HuCCT1 after treatment with IAG933. [file FEB4-14-1873-s002.pdf]

Figure Supplemental 1

A

**Variable**	**LIV31**
FGFR2 Fusion	FGFR2-CCDC6
Anatomic Subtype	iCCA
Age (years)	55
Sex	F

FGFR2, fibroblast growth factor receptor 2; iCCA, intrahepatic cholangiocarcinoma

B

H&E CK7 HepPar1

LIV31

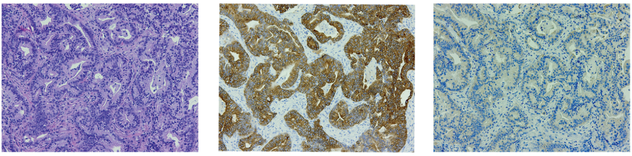

(A) Clinical characteristics of patient derived xenograft utilized for in vivo studies.

(B) Representative photographs hematoxylin and eosin, cytokeratin 7, and HepPar1 staining of LIV31.

Figure Supplemental 2

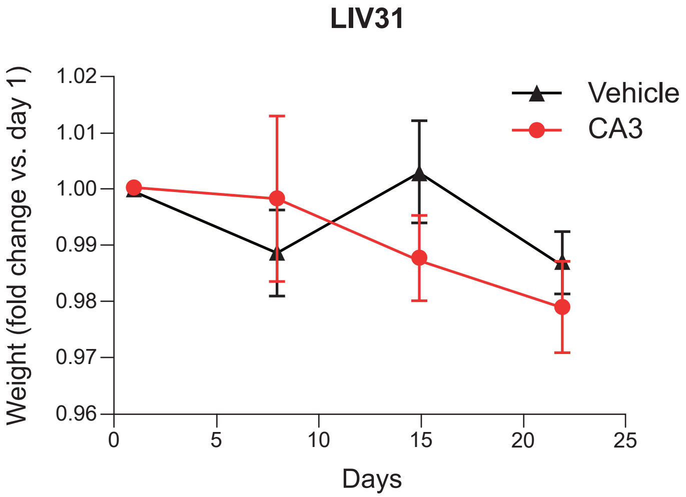

Average weight change from baseline in mice treated with vehicle or CA3 over 3 weeks (n=14). Data represented as mean ± SEM.

Figure Supplemental 3

A

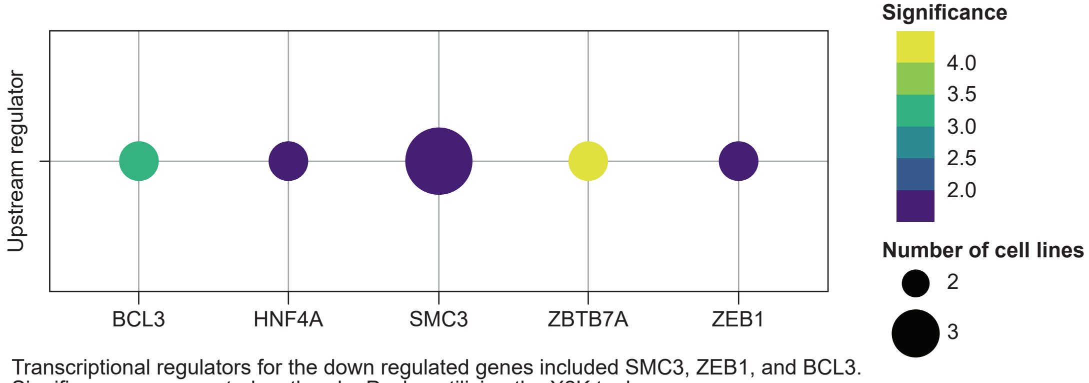

Transcriptional regulators for the down regulated genes included SMC3, ZEB1, and BCL3. Significance was reported as the –logPvalue utilizing the X2K tool.

B

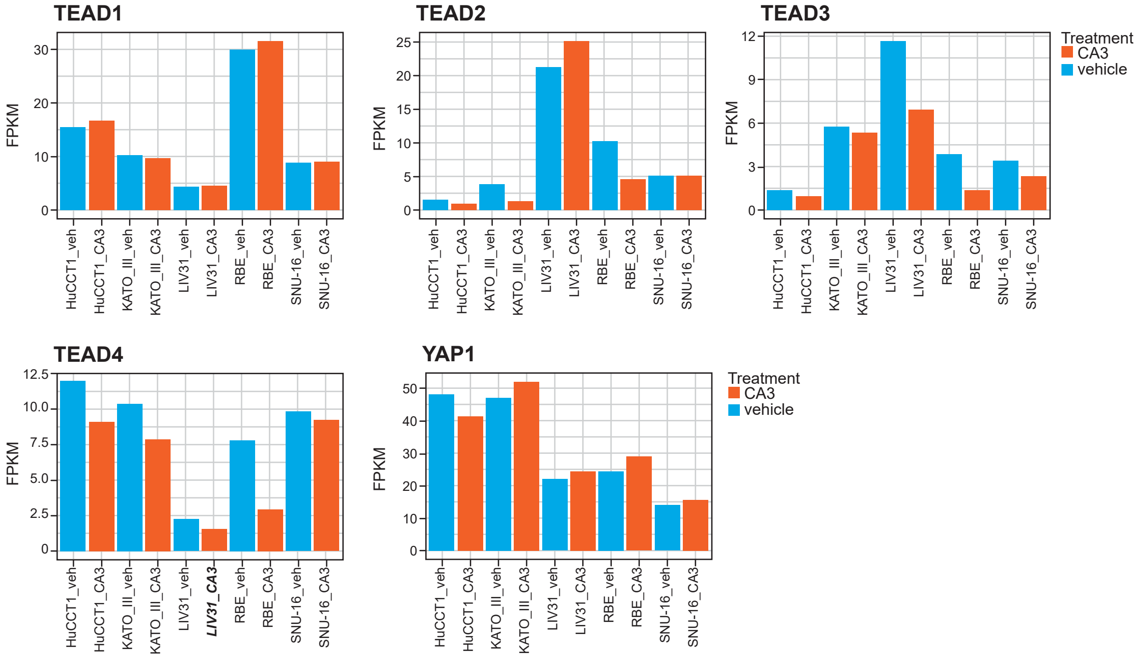

Gene expression of TEAD1-4 and YAP1 utilizing RNA sequencing in cell lines treated with vehicle or CA3. Data represented as fragments per kilobase per million (FPKM).

C

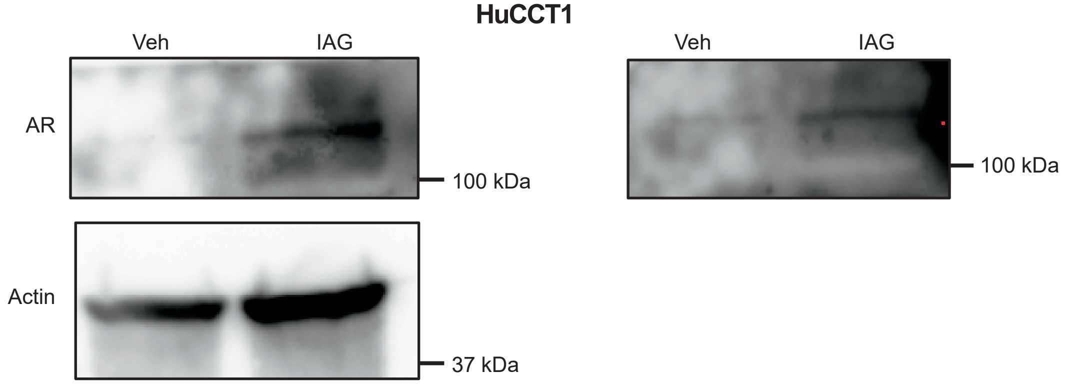

HuCCT1 cell lysates were treated with 1 μM of IAG933, a pan-TEAD inhibitor, for 24 hours and subjected to immunoblot for androgen receptor (AR). AR levels were increased after treatment with IAG933.
