# Supplementary material for: YAP‐TEAD inhibition is associated with upregulation of an androgen receptor mediated transcription program providing therapeutic escape
Source: FEBS Open Bio. 2024 Sep 19;14(11):1873–87. doi: 10.1002/2211-5463.13901 (PMC11532981; doi:10.1002/2211-5463.13901)
Supplement: Supplementary file 2 — Table S1. RT‐PCR primers. [file FEB4-14-1873-s001.docx]

Supplemental Table 1*: RT-PCR primers*

| Gene | Forward sequence (5’ to 3’) . | Reverse sequence (5’ to 3’) |
| --- | --- | --- |
| *Human* |  |  |
| *CTGF* | GCAGCGGAGAGTCCTTCCAG | GGGCCAAACGTGTCTTCCAG |
| *CYR61* | GAGTGGGTCTGTGACGAGGAT | GGTTGTATAGGATGCGAGGCT |
| *NUAK2* | GATGCACATACGGAGGGAGATT | ATCACGATCTTGCTGCTGTTCT |
| *Housekeeping* |  |  |
| *18S* | CGCTTCCTTACCTGGTTGAT | GAGCGACCAAAGGAACCATA |
